# Bidirectional Association between Diabetes and Gout: the Singapore Chinese Health Study

**DOI:** 10.1038/srep25766

**Published:** 2016-05-10

**Authors:** An Pan, Gim Gee Teng, Jian-Min Yuan, Woon-Puay Koh

**Affiliations:** 1 Department of Epidemiology and Biostatistics, and MOE Key Lab of Environment and Health, School of Public Health, Tongji Medical College, Huazhong University of Science and Technology, Wuhan, Hubei, China; 2 Yong Loo Lin School of Medicine, National University of Singapore and National University Health System, Singapore; 3 University Medicine Cluster, Division of Rheumatology, National University of Singapore and National University Health System, Singapore; 4 Division of Cancer Control and Population Sciences, University of Pittsburgh Cancer Institute, Pittsburgh, PA, USA; 5 Department of Epidemiology, University of Pittsburgh Graduate School of Public Health, Pittsburgh, PA, USA; 6 Duke-NUS Graduate Medical School Singapore, Singapore; 7 Saw Swee Hock School of Public Health, National University of Singapore and National University Health System, Singapore

## Abstract

We aimed to prospectively investigate the bidirectional association between type 2 diabetes (T2D) and gout. We analyzed follow-up data from the Singapore Chinese Health Study, when self-reports of diagnosed diabetes and gout were enquired at follow-ups I and II. Individuals who participated in both follow-ups and were free of cardiovascular disease or cancer at follow-up I were included. For T2D to gout (analysis I), prevalent gout were further excluded (final n = 31,137). For gout to T2D (analysis II), prevalent diabetes were excluded (final n = 28,668). Cox regression models were used to estimate relative risks (RRs). In the analysis I, the RR of diabetes to incident gout (682 cases) was 0.77 (95% CI 0.60–0.97). In the analysis II, the RR of gout to incident diabetes (2223 cases) was 1.36 (1.12–1.63), but became insignificant after adjustment for hypertension and BMI (1.00; 0.83–1.21). The gout to diabetes association was modified by BMI (*P*_interaction_ = 0.04) and hypertension (*P*_interaction_ = 0.007), and it was marginally significant in adults with BMI<24 while significant among non-hypertensive participants, but not in their counterparts. In conclusion, our results suggest that diabetes is associated with a lower risk of incident gout, while gout is positively related to diabetes among normal weight and non-hypertensive adults.

Diabetes is a leading risk factor for global disease burden^1^, and it has been estimated that 387 million people had diabetes in 2014, and the number will rise to 592 million by 2035^2^. The prevalence of diabetes in the developing countries has increased dramatically and is fast approaching that in the developed countries. For example, a recent national survey in China has reported a prevalence of diabetes as 11.6% in Chinese adults^3^. Meanwhile, gout is a common arthritis affecting about 5% of middle-aged and elderly population worldwide^4^. The prevalence of gout varies in different populations due to the differences in diagnosis criteria, study population and study design^5^. For example, the US National Health and Nutrition Examination Survey 2007–2008 has reported a prevalence of self-reported gout to be 5.9% in men and 2.0% in women aged >20 years^6^, and a Taiwan survey in 2005–2008 reported 8.2% in men and 2.3% in women^7^.

Both diabetes and gout are associated with increased risk of cardiovascular disease morbidity and mortality^8^,^9^,^10^. Therefore, an association between diabetes and gout in middle-aged and elderly adults has attracted great attention. It has been observed that gout is positively associated with incident diabetes in US adults^11^,^12^ and UK adults^13^. A meta-analysis of 11 cohorts also reported that serum uric acid level is associated with increased risk of developing diabetes^14^. However, a recent prospective study have reported that diabetes is related to a lower risk of incident gout^15^ in the UK general population, and another study reported no significant association between diabetes with incident gout in Chinese adults in Taiwan^16^. The opposite direction of prospective association between diabetes and gout has rarely been reported from a single cohort, except The Health Improvement Network (THIN) in the UK general population^13^,^15^. Therefore, in this study, we aimed to examine the bidirectional association between diabetes and gout in a prospective cohort of middle-aged and older Chinese in Singapore.

## Methods

### Study population

We used data from the Singapore Chinese Health Study (SCHS), a population-based cohort of 63,257 Chinese adults aged 45–74 years at enrolment (1993–1998)^17^. The participants were recruited from two major Chinese dialect groups in Singapore, the Hokkien and Cantonese. Trained interviewers conducted the face-to-face interviews in participants’ homes at recruitment, and obtained the information on demographics, height, weight, tobacco use, physical activity, dietary habits and medical history. Two follow-up interviews were conducted via telephone among surviving participants between 1999 and 2004, and again between 2006 and 2010 to update information on selected lifestyle factors and medical history. The study was approved by the institutional review board of the National University of Singapore, and the study was carried out in accordance with the approved guidelines. All participants have given informed consents.

We used the follow-up I interview (1999–2004) as baseline for our analysis because both gout and diabetes were enquired at this time among 52,322 surviving participants who participated in this interview. During the follow-up II interview (2006–2010), 39,528 participants were re-contacted and information on gout and diabetes was updated.

### Assessment of diabetes and gout

Specifically, at both follow-up interviews, the participants were asked separately if they had been told by doctors that they had diabetes or gout. If the response was “yes”, participants were also asked about the age of first diagnosis. For cases of gout, the interviewers also verified with the participants that the diagnosis was based on joint pain and swelling attributed to reported hyperuricemia by their physicians. For cases of diabetes, a previous validation study was conducted in SCHS, and reported a positive predictive value of 99% when comparing self-reported diabetes status to the hospital-based discharge summary database and a supplementary questionnaire about symptoms, diagnostic tests, and medication use^18^. Furthermore, 5.6% of 2,625 randomly selected participants who reported to be free of diabetes had HbA1c ≥6.5% (47.5 mmol/mol) and thus could be classified as diabetes using the recent diagnostic guidelines^19^. Therefore, 94.4% of participants who reported to be free of diabetes were below the HbA1c cutoff for diabetes. All interviews were tape-recorded and subjected to quality checks.

### Assessment of covariates

At recruitment (1993–1998), participants were asked about their education level, height, weight, tobacco use, physical activity, alcohol intake and medical history. At follow-up I interview (1999–2004), information on body weight, smoking status, alcohol intake and medical history was further updated. Body mass index (BMI) was calculated by weight in kg divided by square of height in meters.

### Statistical analysis

**Figure 1:**
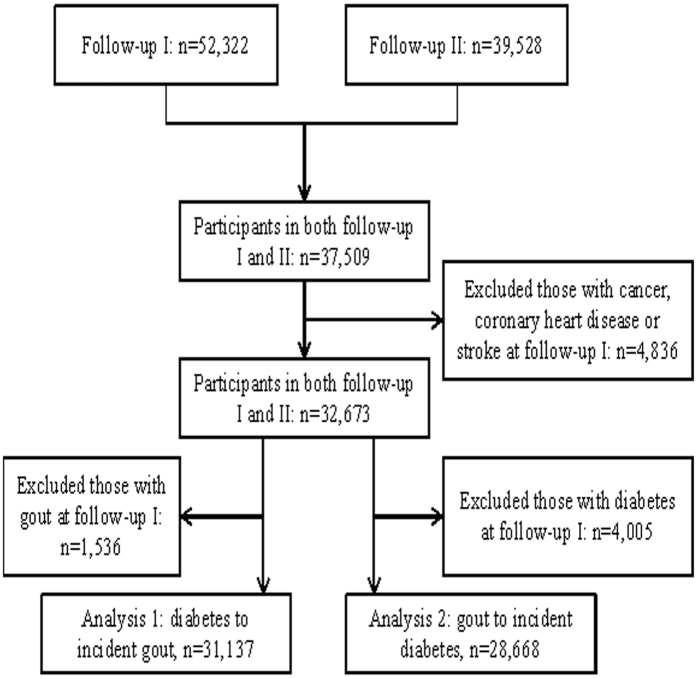
Study flow.

A total of 37,509 individuals participated in both follow-up interviews with complete information on diabetes and gout. We excluded 4,836 participants with history of cancer, coronary heart disease or stroke at follow-up I interview, leaving 32,673 participants for analysis. For the relation of diabetes with incident gout (analysis 1), individuals with history of gout (n = 1536) were further excluded and the final analysis included 31,137 participants. For the analysis of gout and incident diabetes (analysis 2), individuals with prevalent diabetes (n = 4,005) were further excluded and the final analysis included 28,668 participants. The study flow is shown in Fig. 1.

For both analyses, we first compared means or proportions of covariates according to baseline status of diabetes (analysis 1) and gout (analysis 2) at follow-up I interview. For these comparisons, we used χ^2^ tests for categorical variables and t-test for continuous variables. Person-years for each participant were calculated from date of follow-up I interview to the date of reported outcome diagnosis (gout in analysis 1 and diabetes in analysis 2), or the follow-up II interview, whichever occurred first. Cox proportional hazards regression was used to calculate relative risk (RR) and its 95% confidence interval (CI), with adjustment for age (years), sex, dialect (Hokkien/Cantonese), year of follow-up I interview (1999–2001, 2002–2004), educational level (none, primary school, secondary school or higher), moderate physical activity (<0.5, 0.5–3.9, ≥4.0 hours/week), strenuous sports (<0.5, ≥0.5 hours/week) and vigorous work (<0.5, ≥0.5 hours/week), smoking status (never, former, current), alcohol intake (none, monthly, weekly, daily), BMI (<20.0, 20.0–23.9, 24.0–27.9, ≥28.0 kg/m^2^), and self-reported hypertension.

Previous studies have suggested substantial sex differences in prevalence of gout^20^,^21^, and potential sex differences in the relation between diabetes and gout^15^, thus, we also stratified our analyses by sex. Potential interaction tests were explored with categories for BMI (<24.0 and ≥24.0 kg/m^2^) and hypertension (yes and no). We also conducted a 2-year lag sensitivity analysis after excluding early-onset cases. Two-sided *P* value < 0.05 was considered statistically significant, and all analyses were performed with SAS version 9.2 (SAS Institute, Cary, NC).

## Results

The characteristics of the participants at follow-up I interview are shown in Table 1. Compared to non-diabetic participants, diabetic patients were older, had higher BMIs, had lower education levels, and more likely to be former smokers and have hypertension, while less likely to do strenuous sports or vigorous work, to smoke cigarettes or drink alcohol. Compared to individuals without gout, participants with baseline gout had higher BMIs, had higher education levels, had higher prevalence of hypertension, more likely to be male and former smokers, and to be physically active. No difference was found for age.

### Diabetes and risk of incident gout (analysis 1)

After a mean follow-up of 6.9 (SD 1.3) years, 682 participants reported to have incident gout. No significant association was found between diabetes and incident gout in the model without adjustment for BMI and hypertension (RR 0.98; 95% CI 0.77–1.24), while inverse association was evident after adjustment for BMI and hypertension (RR 0.77; 95% CI 0.60–0.97; Table 2). No dose-response relation was observed between duration of diabetes and gout (*P*_trend_ = 0.16; Table 2). The association was not materially changed in the 2-year lag analysis (RR 0.79; 95% CI 0.61–1.00; data not shown).

We further stratified the analysis by sex, BMI and hypertension. The inverse association was seen in men (RR 0.66; 95% CI 0.46–0.96), but not women (RR 0.85; 95% CI 0.62–1.16), although the interaction test was not significant (*P*_*interaction*_ = 0.11). No significant interactions were found with obesity (*P*_*interaction*_ = 0.42) and hypertension status (*P*_*interaction*_ = 0.99), although the association was not significant in some strata because of smaller sample size.

### Gout and risk of incident diabetes (analysis 2)

In the parallel analysis of gout and risk of hypertension, 2,223 participants reported to have incident diabetes during a mean follow-up of 6.2 (SD 1.9) years. Compared to participants without gout, those with gout had a 36% increased risk of developing diabetes (RR 1.36; 95% CI 1.12–1.63); however, the association vanished after adjustment for BMI and hypertension (RR 1.00; 95% CI 0.83–1.21; Table 3). A dose-response association between duration of gout and risk of diabetes was observed (*P*_trend_ < 0.001; Table 3) in the model without adjustment for BMI and hypertension, but not evident after adjustment for those two variables. The association was not substantially different in the 2-year lag analysis (1.04; 0.86–1.25; data not shown).

No significant interaction was found with sex (*P*_*interaction*_ = 0.42) and the association was not significant in either men or women (Table 3). We found significant interactions with BMI (*P*_*interaction*_ = 0.04) and hypertension (*P*_*interaction*_ = 0.007). The association was marginally significant in normal weight participants (RR 1.30; 95% CI 0.96–1.76), but no significant association was found in overweight/obese individuals (0.92; 0.73–1.17). The positive association between gout and incident diabetes was present in non-hypertensive adults (1.38; 1.03–1.85) but not among normotensive individuals (0.85; 0.67–1.09).

## Discussion

In consistent with the UK THIN study, we found an opposite direction of prospective association between diabetes and gout in a cohort of middle-aged and older Chinese in Singapore. Individuals with diabetes are at a lower risk of developing gout. On the other hand, the association between gout and incident diabetes depends on the baseline comorbid BMI and hypertension status: gout was associated with an increased risk of developing diabetes in participants with low-risk profiles (normal weight and/or normotensive individuals), but not in their counterparts.

Many previous cross-sectional studies have reported a strong positive association between hyperuricaemia, gout and metabolic syndrome^22^. Studies have also shown that the prevalence of diabetes among gout patients was as high as 26% in the US adults^23^. However, the comorbidity between gout and diabetes could be because of gout increasing risk of developing diabetes, or diabetes increasing risk of developing gout, or both diseases sharing common risk factors or etiological pathways. Therefore, prospective cohort studies are useful to establish the temporal relations between the two conditions.

Our study found a weak positive association between gout and development of diabetes, but this association disappeared after adjustment for obesity and hypertension status. Several previous prospective studies have shown that gout is associated with increased risk of incident diabetes in Caucasians^11^,^12^,^13^. Choi *et al.*^11^ reported a RR of 1.34 (95% CI 1.09–1.64) in the Multiple Risk Factor Intervention Trial of 11,351 male participants with a high cardiovascular risk profile. In the two studies with both men and women^12^,^13^, a stronger association between gout and incident diabetes was found in women compared to men, although it was significant in both sex. In our study, the association was slightly strong in women compared to that in men (1.15 vs. 0.90), however, neither was significant and the interaction test was not significant either. All three previous studies^11^,^12^,^13^ have adjusted for a number of lifestyle factors and comorbidities, including BMI and hypertension. In our study, the association between gout and incident diabetes disappeared after adjustment for BMI and hypertension, suggesting that these two variables were the major confounding factors in the association. This is possible because obesity and hypertension are major risk factors for both diabetes and gout. As seen from the participants characteristics, patients with gout had substantially higher prevalence of hypertension compared to those without gout (56.0% vs. 33.1%), as well as higher BMI levels (24.6 vs. 23.0 kg/m^2^). We further conducted the stratified analysis by BMI and hypertension status, and found that the association was stronger in normal weight individuals and normotensive participants, and the interaction tests were significant. This suggested that gout might be an independent risk factor for developing diabetes among low-risk profile individuals, or the risk induced by gout in high-risk participants could be shadowed by obesity and hypertension, both are strong risk factors for diabetes. In the study by Choi *et al.*^11^, the authors reported no significant interactions with baseline obesity and hypertension; however, no data of the stratified analyses were reported, and no other studies have tested the interactions with obesity and hypertension status. Therefore, our results could also be a chance finding and need to be further tested.

Our results suggest that gout and diabetes may share the common etiological pathway of abnormal metabolic status, characterized by high blood pressure, obesity, and excess uric acid. A meta-analysis of 11 cohorts also reported that serum uric acid level is associated with increased risk of developing diabetes^14^, including studies in Chinese populations^24^,^25^. Gout is a common arthritis caused by deposition of monosodium urate crystals within joints due to hyperuricaemia^26^. Therefore, hyperuricaemia may causally lead to both gout and diabetes. However, we did not have data on serum uric acid levels, and thus could not evaluate whether hyperuricaemia was the common risk factor for both diabetes and gout. Uric acid may worsen insulin resistance by inhibiting the bioavailability of nitric oxide, which is crucial for insulin-stimulated glucose uptake^27^,^28^. Although uric acid is a major antioxidant and can help protect against free-radical oxidative damage^29^, the high circulating level may also serve as a marker of excessive free radicals and oxidative stress. Finally, hyperuricaemia is closely related to other cardiometabolic risk factors (obesity, hypertension, metabolic syndrome and inflammation)^30^, all of them are causally related to increased risk of diabetes and gout. In our analysis, the association between gout and diabetes risk disappeared after adjustment for obesity and hypertension.

As for the relation between type 2 diabetes and risk of new-onset gout, the findings have been inconsistent. A recent large study in 132,556 Chinese adults found no significant association in men (RR 0.85; 95% CI 0.67–1.07) and women (RR 1.15; 95% CI 0.83–1.60)^16^, while the study in the UK THIN nested case-control study reported a stronger inverse association in men (RR 0.61; 95% CI 0.57–0.66) but not significant in women (RR 0.91; 95% CI 0.81–1.02)^15^. Our study findings are highly consistent with the UK study: we observed a RR of 0.66 (95% CI 0.46–0.96) in men and 0.85 (95% CI 0.62–1.16) in women. The interaction test was not significant (*P* = 0.11), probably because of our small sample size. In a recent study among type 2 diabetic patients using the UK-based Clinical Practice Research Datalink, Bruderer and colleagues^31^ reported that increased HbA1c levels was associated with a decreased risk of incident gout. These findings seem counterintuitive given the strong association between hyperuricaemia and metabolic syndrome^22^,^32^,^33^,^34^,^35^,^36^, a pre-diabetes status. Rodrigues *et al.*^15^ proposed that the strong association exists during pre-diabetes status but not after type 2 diabetes develops, and the reduced risk of gout might be due to the uricosuric effect of glycosuria or the impaired inflammatory response observed in diabetes. Some previous studies have consistently shown that diabetic patients had lower serum uric acid levels compared with normal individuals^37^,^38^,^39^,^40^,^41^. The Atherosclerosis Risk in Communities Study also found that every additional 5 years’ duration of diabetes was associated with a 0.10 mg/dL lower uric acid level among participants with diabetes^42^. In our study, we did not see the steady reduction of risk of incident gout with more years of diabetes duration, but the sample size was small.

To the best of our knowledge, our study is the first to investigate the bidirectional association between gout and diabetes in Chinese population. We have used the data from a population-based prospective cohort with a relatively long-term follow-up. We have tried to control multiple potential risk factors for gout or diabetes in our analysis. In addition, participants with prevalent history of cancers, coronary heart disease, and stroke were excluded from the analysis since they could be associated with gout or diabetes.

Several limitations in our study warrant attention. First, both diabetes and gout were self-reported, and we did not collect detailed information on the treatment of the diseases. The study by Bruderer *et al.*^31^ did not find significant associations between diabetes medications (insulin, metformin, and sulfonylureas) and risk of incident gout. Thus, the lower risk of gout associated with diabetes might not be explained by medications. Second, we did not have information on the serum uric acid levels, and thus could not test whether the gout to diabetes association was due to the relatively higher blood levels of uric acid. Studies have shown that serum uric acid level was associated with increased risk of developing diabetes^14^, thus gout and diabetes might share the common cause of hyperuricaemia. Third, validation using drug prescription data and medical records for gout was not feasible in our study. Although we had trained our interviewers to further enquire if the joint pain and swelling from gout was attributed to reported hyperuricemia by their physicians in order to increase the accuracy of self-reported gout, misclassification is possible. Using this case definition, we have previously shown the association between gout and mortality risk^43^, as well as between diet and risk of incident gout from this cohort^44^. As with large population-based studies, it is not feasible to require the presence of intra-articular urate crystals or tophus as the gold standard for diagnosis of gout. Some other population-based cohorts have shown that self-reported gout could have moderate to good reliability and sensitivity, and therefore be an adequate proxy for the actual case in epidemiological studies^45^,^46^,^47^. As for diabetes, we have conducted a validation study of self-reported diabetes and found a very high positive predictive value, suggesting that the self-reported diabetes cases were more likely to be true cases. However, undiagnosed gout or diabetes was still possible in the study population, and this might attenuate the associations because undiagnosed cases were classified as non-exposed group and thus narrowed the differences between exposed and non-exposed groups. Furthermore, surveillance bias due to disease diagnosis is also a possible explanation for our findings, since people with diabetes or gout may be more likely to see the doctors and get their blood glucose or serum uric acid levels measured. However, this surveillance bias could not explain the opposite direction of the associations between the two diseases. We have also applied the 2-year lag analysis and the results remained unchanged. In addition, the sample size was limited in some subgroups, and future large studies are still needed. Finally, unmeasured and residual confounding (such as metabolic syndrome parameters, inflammatory factors, body composition etc.) is possible in the study and may influence the accuracy of the observed associations.

In conclusion, our findings suggest that type 2 diabetic patients are at a lower risk of developing gout. On the other hand, individuals with gout have a higher future risk of diabetes, particularly among those who are normal weight or free of hypertension. The opposite directions of the prospective association between the two diseases warrant further validation in other populations. The results also indicate that the etiologic mechanisms are complex and different for the two directions, and future studies are needed to explore the underlying pathways, which are important for the understanding of the pathophysiology and treatment of gout and diabetes.

## Additional Information

**How to cite this article**: Pan, A. *et al.* Bidirectional Association between Diabetes and Gout: the Singapore Chinese Health Study. *Sci. Rep.* **6**, 25766; doi: 10.1038/srep25766 (2016).

## Figures and Tables

**Table 1:** Characteristics of cohort participants at follow-up I interview (1999–2004) in the two analyses: The Singapore Chinese Health Study.

Characteristics	Analysis 1: diabetes to incident gout			Analysis 2: gout to incident diabetes		
	Participants with baseline diabetes	Participants without diabetes	*P* value*	Participants with baseline gout	Participants without gout	*P* value*
Number of participants	3849 (12.4)	27288 (87.6)		1194 (4.2)	27474 (95.8)	
Age, y	62.1 ± 7.2	60.3 ± 7.3	<0.001	60.3 ± 7.2	60.3 ± 7.3	0.82
Body mass index, kg/m^2^	24.2 ± 3.5	22.9 ± 3.4	<0.001	24.6 ± 3.5	23.0 ± 3.4	< 0.001
Male	1529 (39.7)	10856 (39.8)	0.94	744 (62.3)	10950 (39.9)	< 0.001
Cantonese Dialect	1871 (48.6)	13589 (49.8)	0.17	580 (48.6)	13688 (49.8)	0.40
Hypertension	2393 (62.2)	9004 (33.0)	< 0.001	668 (56.0)	9103 (33.1)	< 0.001
Education level			<0.001			< 0.001
No	1075 (27.9)	6258 (22.9)		151 (12.7)	6285 (22.9)	
Primary school	1770 (46.0)	12204 (44.7)		510 (42.7)	12284 (44.7)	
Secondary school or higher	1004 (26.1)	8826 (32.3)		533 (44.6)	8905 (32.4)	
Smoking status			<0.001			< 0.001
Never	2773 (72.0)	19921 (73.0)		772 (64.7)	20045 (73.0)	
Former	604 (15.7)	3267 (12.0)		263 (22.0)	3303 (12.0)	
Current	472 (13.3)	4100 (15.0)		159 (13.3)	4126 (15.0)	
Moderate physical activity			0.98			< 0.001
<0.5 hours/week	3016 (78.4)	21356 (78.3)		854 (71.5)	21497 (78.2)	
0.5–3.9 hours/week	545 (14.2)	3864 (14.2)		218 (18.3)	3896 (14.2)	
≥4 hours/week	288 (7.5)	2068 (7.6)		122 (10.2)	2081 (7.6)	
Weekly strenuous sports			<0.001			< 0.001
<0.5 hours/week	3602 (93.6)	24996 (91.6)		1025 (85.8)	27172 (91.6)	
≥0.5 hours/week	247 (6.4)	2292 (8.4)		169 (14.2)	2302 (8.4)	
Weekly vigorous work			<0.001			0.01
<0.5 hours/week	3582 (93.1)	24951 (91.4)		1066 (89.3)	25114 (91.4)	
≥0.5 hours/week	267 (6.9)	2337 (8.6)		128 (10.7)	2360 (8.6)	
Alcohol intake			<0.001			0.03
Abstainers	3579 (93.0)	24202 (88.7)		1029 (86.2)	24357 (88.7)	
Weekly drinkers	222 (5.8)	2264 (8.3)		119 (10.0)	2285 (8.3)	
Daily drinkers	48 (1.3)	822 (3.0)		46 (3.9)	832 (3.0)	

Values are shown in mean ± standard deviation or n (%).

^*^P value was calculated by t-test for continuous variables and χ^2^ test for categorical variables.

**Table 2:** Hazard ratios (95% confidence intervals) for risk of gout according to diabetes status: The Singapore Chinese Health Study (1999–2010).

	Cases/person-years	Model 1	Model 2
Baseline (follow-up I) diabetes status			
No	603/188390	1.00 (ref)	1.00 (ref)
Yes	79/25852	0.98 (0.77–1.24)	0.77 (0.60–0.97)
Stratified by sex*			
Men			
No	302/74002	1.00 (ref)	1.00 (ref)
Yes	32/10117	0.79 (0.55–1.14)	0.66 (0.46–0.96)
Women			
No	301/114387	1.00 (ref)	1.00 (ref)
Yes	47/15735	1.14 (0.83–1.55)	0.85 (0.62–1.16)
Stratified by BMI category*			
Normal weight			
No	306/125538	1.00 (ref)	1.00 (ref)
Yes	34/13916	0.98 (0.69–1.40)	0.82 (0.57–1.17)
Overweight/obesity			
No	297/62852	1.00 (ref)	1.00 (ref)
Yes	45/11936	0.81 (0.59–1.11)	0.73 (0.53–1.01)
Stratified by hypertension*			
No hypertension			
No	299/126550	1.00 (ref)	1.00
Yes	19/9831	0.83 (0.52–1.33)	0.76 (0.47–1.20)
With hypertension			
No	304/61840	1.00 (ref)	1.00
Yes	60/16021	0.78 (0.59–1.03)	0.76 (0.57–1.00)
Duration of diabetes			
No diabetes	603/188390	1.00 (ref)	1.00 (ref)
0.1–4.9 years	23/7822	0.98 (0.64–1.48)	0.73 (0.48–1.11)
5.0–9.9 years	20/6449	0.98 (0.63–1.53)	0.78 (0.50–1.21)
≥10.0 years	28/8638	1.02 (0.70–1.49)	0.86 (0.59–1.26)
*P* for trend†		0.98	0.16

Model 1: adjusted for age, sex, dialect, year of interview, educational level, moderate physical activity, strenuous sports, vigorous work, smoking status, and alcohol use;

Model 2: model 1 plus body mass index ( < 20.0, 20.0–23.9, 24.0–27.9, ≥ 28 kg/m^2^), and history of hypertension at follow-up I; but not the variable itself in the stratified analysis.

^*^The *P* for interaction was 0.11 for sex, and 0.42 for BMI category ( < 24 and ≥ 24 kg/m^2^), 0.99 for hypertension in model 2.

^†^*P* for trend was calculated by treating the categorical variable of duration of hypertension as a continuous variable. 415 participants did not answer the question of age of diabetes diagnosis, and among them 8 developed gout.

**Table 3:** Hazard ratios (95% confidence intervals) for risk of diabetes according to gout status: The Singapore Chinese Health Study (1999–2010).

	Cases/person-years	Model 1	Model 2
Baseline (follow-up I) gout status			
No	2103/186162	1.00 (ref)	1.00 (ref)
Yes	120/8064	1.36 (1.12–1.63)	1.00 (0.83–1.21)
Stratified by sex*			
Men			
No	806/73438	1.00 (ref)	1.00 (ref)
Yes	67/4982	1.24 (0.96–1.59)	0.91 (0.70–1.17)
Women			
No	1297/112725	1.00 (ref)	1.00 (ref)
Yes	53/3081	1.48 (1.13–1.95)	1.15 (0.87–1.52)
Stratified by BMI category*			
Normal weight			
No	981/124564	1.00 (ref)	1.00 (ref)
Yes	44/3751	1.53 (1.13–2.08)	1.30 (0.96–1.76)
Overweight/obesity			
No	1122/61598	1.00 (ref)	1.00 (ref)
Yes	76/4313	0.99 (0.79–1.26)	0.92 (0.73–1.17)
Stratified by hypertension			
No hypertension			
No	1033/125409	1.00 (ref)	1.00 (ref)
Yes	48/3578	1.69 (1.26–2.26)	1.38 (1.03–1.85)
With hypertension			
No	1070/60754	1.00 (ref)	1.00 (ref)
Yes	71/4485	0.94 (0.74–1.19)	0.85 (0.67–1.09)
Duration of gout			
No gout	2103/186162	1.00 (ref)	1.00 (ref)
0.1–4.9 years	52/4002	1.16 (0.88–1.53)	0.87 (0.66–1.15)
5.0–9.9 years	32/1836	1.59 (1.12–2.26)	1.14 (0.80–1.62)
≥10.0 years	35/2197	1.51 (1.08–2.11)	1.11 (0.79–1.56)
*P* for trend†		<0.001	0.62

Model 1: adjusted for age, sex, dialect, year of interview, educational level, moderate physical activity, strenuous sports and vigorous work, smoking status, and alcohol use; but not sex in the sex-stratified analysis;

Model 2: model 1 plus body mass index (<20.0, 20.0–23.9, 24.0–27.9, ≥28 kg/m^2^), and history of hypertension at follow–up I; but not the variable itself in the stratified analysis.

^*^The *P* for interaction was 0.19 for sex, 0.04 for BMI category (<24 and ≥24 kg/m^2^), and 0.007 for hypertension in model 2.

^†^*P* for trend was calculated by treating the categorical variable of duration of gout as a continuous variable. Four participants did not answer the question of age of gout diagnosis, and among them 1 developed diabetes.
